# The Chain Ratio Estimator and Regression Estimator with Linear Combination of Two Auxiliary Variables

**DOI:** 10.1371/journal.pone.0081085

**Published:** 2013-11-18

**Authors:** Jingli Lu

**Affiliations:** College of Sciences, Inner Mongolia University of Technology, Hohhot, China; Memorial Sloan Kettering Cancer Center, United States of America

## Abstract

In sample surveys, it is usual to make use of auxiliary information to increase the precision of the estimators. We propose a new chain ratio estimator and regression estimator of a finite population mean using linear combination of two auxiliary variables and obtain the mean squared error (MSE) equations for the proposed estimators. We find theoretical conditions that make proposed estimators more efficient than the traditional multivariate ratio estimator and the regression estimator using information of two auxiliary variables.

## Introduction

The use of supplementary information provided by auxiliary variables in survey sampling was extensively discussed [1]–[10]. The ratio estimator and regression estimator are among the most commonly adopted estimators of the population mean or total of study variable of a finite population with the help of two auxiliary variables when the correlation coefficient between the two variables is positive. It is well known that these estimators are more efficient than the usual estimator of the population mean based on the sample mean of a simple random sampling.

In this study, we proposed a new chain ratio estimator and regression estimator using linear combination of two auxiliary variates, and obtain the mean squared error (MSE) equations for the two proposed estimators. The proposed estimators, the traditional multivariate ratio estimator and the regression estimator using information of two auxiliary variables were compared at theoretical conditions. And we obtained the satisfactory results.

## Materials and Methods

### The existed estimators

The classical ratio estimator and regression estimator for the population mean 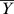 of the variate of interest *y* using one auxiliary information are defined by 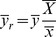
(1)

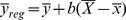
(2)where it is assumed that the population mean 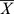 of the auxiliary variate *x* is known.

Here
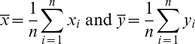
(3)where *n* is the number of units in the sample[11], and 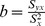 is the regression coefficient for of *Y* on *X*. 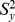
and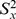 are the population variances of the *y_i_* and *x_i_*, respectively. 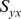 is the population covariance between *y_i_* and *x_i_* [11].

The MSE of the classical ratio estimator is
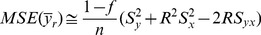
(4)where 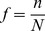; *N* is the number of units in the population; 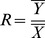 is the population ratio, 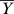
and 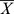 are the population means of the *y_i_* and *x_i_* respectively.

The MSE of the regression estimator is
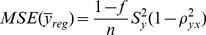
(5)where 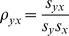 is the population correlation coefficient between 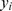 and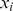.

Kadilar and Cingi[12] proposed the chain ratio estimator using one auxiliary information for 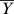 as 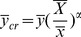
(6)where 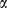 is real number.

MSE of this estimator is given as follows:
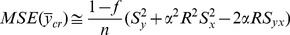
(7)


The traditional multivariate ratio estimator and regression estimator using information of two auxiliary variables *x*_1_ and *x*_2_ to estimate the population mean, 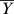 [13], as follows:
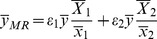
(8)

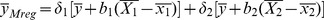
(9)where 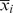 and 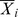
(*i* = 1,2) denote respectively the sample and the population means of the variable *x_i_* (*i* = 1,2); 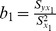 and 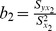 are the regression coefficients of on 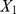 and on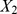, respectively, here 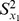 and 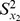 are the variances of 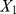 and 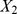, respectively, and 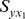 and 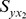 are the covariances between *Y* and 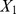, *Y* and 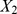, respectively.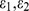,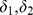 are the weights that satisfy the condition, respectively: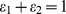 and 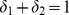.

The MSE of this traditional multivariate ratio estimator is given by 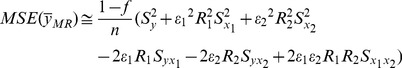
(10)where 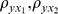 and 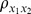
denote the correlation coefficient between *Y* and *X*_1_, *Y* and *X*_2_, *X*_1_ and *X*_2_ respectively. 
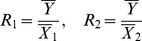
.

The optimum values of 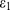 and 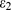 are given by 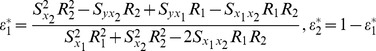



The minimum MSE of 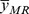 can be shown to be:
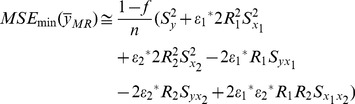
(11)


The MSE of this traditional multivariate regression estimator is given by
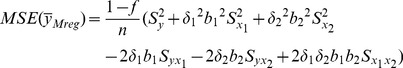
(12)


The optimum values of 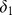 and 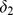 are given by 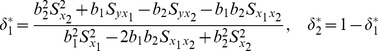



The minimum MSE of 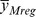 can be shown to be:
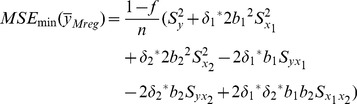
(13)


### The suggested estimators

We propose the multivariate chain ratio estimator and regression estimator using linear combination of two auxiliary variables as follows: 
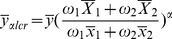
(14)

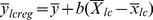
(15)where 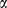 is a arbitrary constant, 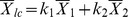,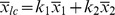,and 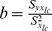 is the regression coefficient on 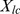.



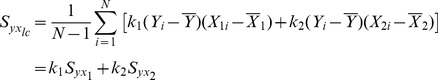


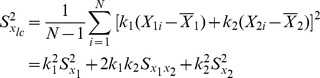




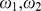
 and 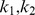 are weights that satisfy the condition: 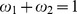 and 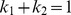.

The MSE of this new multivariate ratio estimator is given by 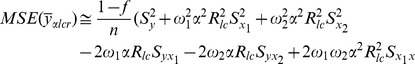
(16)where 
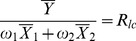


The optimum values of 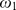 and 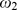 are given by 
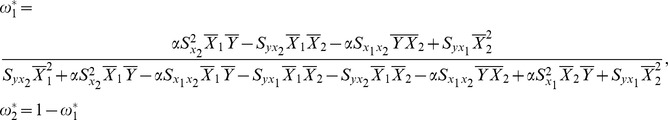



The minimum MSE of 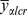 can be shown to be:
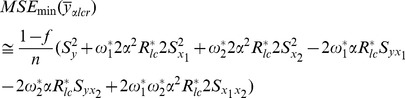
(17)where 
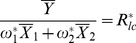


The MSE of this new multivariate regression estimator is given by
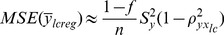
(18)


Where 
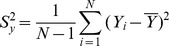
,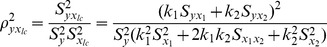.

The optimum values of 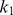 and 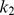 are given by 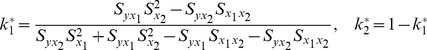



The minimum MSE of 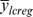 can be shown to be:
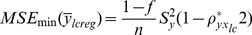
(19)


Where 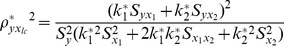


### Efficiency comparison

We compare the MSE of the proposed multivariate ratio estimator using information of two auxiliary variables given in Eq. (17) with the MSE of traditional multivariate ratio estimator using information of two auxiliary variables given in Eq.(11) as follows:
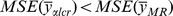


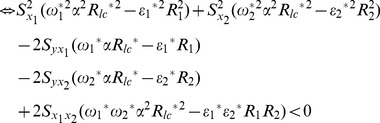
(20)


We compare the MSE of the proposed regression estimators given in Eq. (19) with the MSE of the traditional multivariate regression estimator using information of two auxiliary variables given in Eq.(13) as follows:
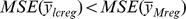


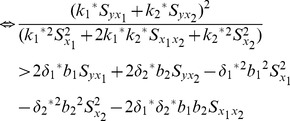
(21)


### Numerical illustration

The comparison among these estimators is given by using a data set whose statistics are given in Table 1 [14]. we apply the traditional multivariate ratio estimator and regression estimator using information of two auxiliary variables, given in Eqs.(8) and (9) and proposed chain ratio estimator and regression estimator of a finite population mean using linear combination of two auxiliary variables, given in Eqs. (14) and (15), to data whose statistics are given in Table 1. We assume to take the sample size *n* = 70, from *N* = 180 using SRSWOR. The MSE of these estimators are computed as given in Eqs.(11), (13), (17) and (19).

**Table 1:** Data Statistics.

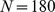	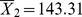	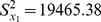	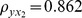
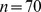	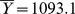	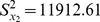	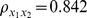
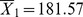	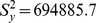	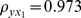	

## Results and Discussion

MSE values of the traditional multivariate ratio estimator and regression estimator using information of two auxiliary variables and proposed chain ratio estimator and regression estimator using linear combination of two auxiliary variables can be seen in Table 2.

**Table 2:** MSE Values of Estimators.

Estimators	MSE
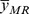	0.1576
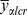	0.1574 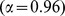
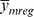	0.1766
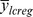	0.1574

From Table 2, we notice that our proposed chain ratio estimator using linear combination of two auxiliary variables 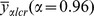 is more efficient than traditional multivariate ratio estimator using information of two auxiliary variables and our proposed regression estimator using linear combination of two auxiliary variables 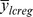 is more efficient than traditional multivariate regression estimator using information of two auxiliary variables. We examine the conditions for this data set, 
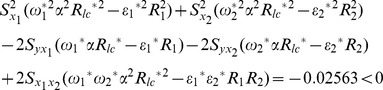


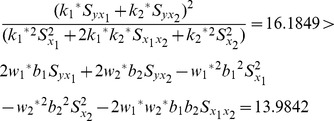



The result shows that the condition (20) and condition (21) are satisfied. Therefore, we suggest that we should apply the proposed estimators to this data set.

## Conclusions

We develop a new chain ratio estimator and a new regression estimator of a finite population mean using two auxiliary variables and theoretically show that the proposed estimators are more efficient than the traditional ratio estimator and traditional regression estimator using two auxiliary variables in certain condition.
